# Treatment of Exposed Pulps

**Published:** 1876-02

**Authors:** D. D. Peabody


### ARTICLE VIII.

### *Treatment of Exposed Pulps.*

BY D. D. PEABODY.

Read before the Eighth District Dental Society, Buffalo, N. Y., May, 1875.

The dental pulp may be said to be exposed when the covering which has been provided for its protection has been by any means removed, permitting the approach and action of external influences. Occasionally, the removal of such protection is followed by no serious results for months, and even years; but, as we have learned by experience, the great majority of such exposures are speedily succeeded by the most intense pain, calling into requisition all our skill, as well as patience, for its alleviation, and the ultimate restoration of the tooth to its normal condition.

Let us look at the nature of the agencies most active in causing irritation of the pulp. They are numerous; but we will only mention three: chemical, thermal, and mechanical. The first two are most frequently found in operation in cavities of decay where sufficient enamel remains to prevent the ingress of hard substances in close proximity to the pulp, but where cold and hot fluids and chemical agents in solution readily find their way, and are generally the cause of the first alarm. Such are usually found between molars and bicuspids; while those oftener affected by mechanical means alone, are in the crown cavities of molars, and the worn and concaved apices of canines and incisors.

Let us consider first the simplest form of exposure, existing where the grinding surfaces have been worn by attrition until mastication has become painful. In such teeth there is usually no caries. Nearly always, the pulp has protected itself by the deposit of osteo-dentine from being completely uncovered, but not from the action of pressure on the fluids contained in the minute dentinal tubuli sufficient to be transmitted to the pulp, and thus cause pain. My only treatment for this difficulty has been complete dryness, and the thorough application of a moistened pencil of fused nitrate of silver to the sensitive surface, which should then be allowed to dry for ten or fifteen minutes. Thus far I have found one application to be sufficient. The next degree of difficulty in treatment is that form found in the approximal and distal cavities of incisors and canines and the crown cavities of molars; while those most difficult of access to the operator, and probably for this reason, the last to yield to curative treatment, are those in the approximal, distal and buccal surfaces of molars and buccal surfaces of molars and bicuspids. The first thing to be done is to ascertain the exact condition of the interior of the cavity, an unobstructed view of which may be best obtained, if at all, after the application of the rubber-dam. After the cavity is dried as much as may be by absorbents and warm air, the carious portion should be carefully removed with sharp excavators, exercising care to draw the instrument, not toward the inflamed organ, nor parallel to it, but directly away from it. After this is accomplished, if there is no discharge of serum or pus, and the most delicate touch of the instrument over the exposed portion causes slight pain, we may conclude that the pulp, though highly inflamed, has not been to any extent destroyed by disease. If the pain is accompanied by severe throbbing, an incision with a very sharp and thin lance is followed by almost immediate relief from pain. After the flow of blood has ceased—and the freer the flow the better—dry the cavity gently but thoroughly, and moisten whole interior with absolute alcohol. This may be readily obtained by the addition of the dried carbonate of potassa to strong alcohol, in the proportion of two drachms to the fluid ounce, and it will be found invaluable in this connection. Prepare the edges of the walls as if for final plugging. If there is a recurrence of the pain after depletion, tincture aconite one drachm, to one ounce chloroform may be used to good advantage. When complete relief from pain has been obtained, a few minutes rest from further treatment is advisable, in order to give complete assurance of the success of the foregoing; after which, if all remains quiet, the cavity is in readiness for the insertion of some filling to protect the work already accomplished. Now the question arises, what shall this be? There are various methods advanced by different operators, but I will only speak briefly of my own. After reading carefully the different views of many writers, and witnessing the operations of others, I have used in succession a thin paste of oxy-chloride of zinc, which I now regard as extremely dangerous; a varnish of Canada balsam in chloroform, followed by the oxy chloride which is little better; and gutta percha varnish, which is liable to fail from shrinking and drawing away from the walls. Though the last two are far preferable to the first, yet so much pain follows the insertion of the oxy-chloride, that for some minutes it would seem as if the last state of the pulp was worse than the first. Neither of these methods have proved entirely satisfactory. If practicable, I prefer to leave the osteo-plastic capping or filling *in situ* for two, four and even six months before inserting the final plug. This form of treatment I have practiced for about five years; and, prompted by curiosity, have removed several cappings to assure myself of results, which have been in most cases favorable; still I dare not assert that this course is by any means safe or certain. For a few months past I have found far more satisfaction to myself, and doubtless to the patient, from the employment of a varnish composed of gum copal one drachm, and pure sulphuric ether one ounce. Apply freely to the dried surface of the dentine and pulp, exercising caution that it be not allowed to pass outside the walls of the cavity. Dry with warm air and apply a second time, which will be found sufficient to cover the surface with a hard coating—closely adherent, non-conducting, and non-irritant. Over this, place a soft coating of Guillois' Cement, and allow it to harden; then fill the cavity with the same material, mixed as stiff as it can be manipulated, allow it to dry for ten minutes, and coat with sandarac varnish.—*Dental Advertiser.*

---

